# Postmarketing Analysis of Misuse, Abuse, and Diversion of Xtampza ER

**DOI:** 10.1093/pm/pnaa272

**Published:** 2020-10-23

**Authors:** Stevan Geoffrey Severtson, Scott E D Kreider, Elise C Amioka, Zachary R Margolin, Janetta L Iwanicki, Richard C Dart

**Affiliations:** Rocky Mountain Poison & Drug Safety (RMPDS), A Division of Denver Health, Denver, Colorado, USA

**Keywords:** Pain Management, Abuse Deterrent Formulations, Extended-Release Opioid Analgesics, Oxycodone, Prescription Drug Abuse

## Abstract

**Objective:**

To evaluate abuse, misuse, and diversion of Xtampza ER, an extended-release (ER) abuse-deterrent formulation (ADF) of oxycodone.

**Methods:**

Abuse, misuse, and diversion of Xtampza ER were assessed using Researched Abuse, Diversion and Addiction-Related Surveillance (RADARS) System data sources. Xtampza ER was compared with immediate-release (IR) oxycodone, other ADF ER products combined, and non-ADF ER products combined.

**Results:**

Xtampza ER prescriptions increased 50-fold during the study period. In contrast, cases from poison centers, substance abuse treatment centers, and diversion were infrequent and did not increase. Adjusted for prescriptions dispensed, poison center exposures were greater for IR oxycodone (rate ratio [RR] = 2.3, *P* = 0.008), other ADF ER opioids (RR = 5.2, *P* < 0.001), and non-ADF ER opioids (RR = 2.5, *P* = 0.004) than for Xtampza ER. In Treatment Center Programs Combined, past-month abuse prevalence for other ADF ER opioids (odds ratio [OR] = 7.4, *P* < 0.001) and non-ADF ER opioids (OR = 2.0, *P* = 0.002) was greater than Xtampza ER; IR oxycodone was not significantly different (OR = 1.2, *P* = 0.349). In the Drug Diversion Program, rates for IR oxycodone (RR = 3.7, *P* = 0.003), other ADF ER opioids (RR = 4.2, *P* = 0.002), and non-ADF ER opioids (RR = 3.4, *P* = 0.007) were greater than Xtampza ER. Adjustment using morphine equivalents provided similar results, except that IR oxycodone in Treatment Center Programs Combined became higher than Xtampza ER. Nonoral abuse cases involving Xtampza ER were infrequent; Web monitoring data support findings that Xtampza ER is difficult to abuse nonorally.

**Conclusion:**

Xtampza ER abuse, misuse, and diversion and tampering are low relative to other prescription opioid analgesics. Abuse and diversion did not increase over the study period.

## Introduction

Opioid analgesics are an established treatment for acute and chronic pain. However, inappropriate use of these medications is common and associated with serious risks. In 2018, an estimated 2.9 million individuals in the United States used a prescription pain reliever nonmedically in the past month [1]. In 2017, more than 17,000 individuals died from an overdose involving prescription opioids [2]. 

Tampering or manipulating pills to facilitate nonoral use is common among individuals who abuse opioids, with half of respondents who reported abusing opioids in the past three months indicating that they tampered with opioid medications [3]. Immediate-release (IR) opioid analgesics are abused more often than extended-release (ER) products [4]; however, ER opioids may be more desirable (and dangerous) to manipulate because bypassing the controlled-release mechanism provides a greater amount of drug to be absorbed more quickly. Tampering with opioid analgesics is associated with greater risk of serious health events, including death [5].

In April 2015, the US Food and Drug Administration (FDA) released final guidance for manufacturers of opioids to demonstrate abuse deterrence of specific products [6]. The FDA guidance allows product labeling describing the effectiveness of these technologies. The guidance provides a framework for evaluating abuse deterrence through four study categories. Category 1 (laboratory in vitro manipulation and extraction), category 2 (pharmacokinetic), and category 3 (clinical abuse potential) studies evaluate abuse-deterrent properties in premarket, nonclinical, and clinical settings. Category 4 (postmarket) studies evaluate whether a product with abuse-deterrent labeling based on premarket studies results in meaningful reductions in abuse, misuse, and other adverse outcomes after the product is released to market. There are currently seven marketed products, including six ER products, with approved labeling describing abuse-deterrent properties based on premarket data. However, no opioid product currently includes labeling demonstrating reduced abuse in the postmarketing setting (category 4).

Measuring nonmedical use of a prescription opioid product in the community setting presents methodological challenges. These behaviors are stigmatizing and illegal. A common strategy for evaluation of abuse and related behaviors and outcomes in the postmarket setting is the use of multiple data sources [7]. The goal is to measure illicit behaviors when the individual chooses to reveal their behavior, such as calling a poison center or entering treatment for substance abuse. This multifaceted or “mosaic” approach has been used to evaluate trends of prescription opioid and heroin use [8], effectiveness of an ER oxycodone formulation with abuse-deterrent labeling [9], effectiveness of interventions intended to reduce prescription opioid abuse/misuse [10], and the abuse liability of opioid analgesics in the years after their initial marketing [11–13].

Xtampza ER (Collegium Pharmaceutical, Stoughton, MA, USA) is an oxycodone analgesic with properties intended to discourage tampering. First marketed in 2016, Xtampza ER was granted abuse-deterrent labeling with respect to oral, nasal, and intravenous routes of administration based upon data obtained in the premarket setting.

The purpose of this study is to measure the relative abuse, misuse, and diversion of prescription opioid analgesics including Xtampza ER in the postmarket setting. We assess whether Xtampza ER abuse, misuse, and diversion have changed since launch and if these outcomes and price in the illegal market are lower than other opioid analgesics. We also characterize attempts at tampering by evaluating Web forums, discussion boards, and poison center case notes.

## Methods

The Researched Abuse, Diversion and Addiction-Related Surveillance (RADARS) System utilizes data collected from multiple sources to assess prescription drug use. Data from the following RADARS System programs were included: 1) Poison Center Program, 2) Drug Diversion Program, 3) Treatment Center Programs Combined (Opioid Treatment Program and the Survey of Key Informants’ Patients Program), 4) the StreetRx Program, and 5) the Web Monitoring Program. The analysis period was the first three years after initial marketing of Xtampza ER: July 1, 2016, through June 30, 2019.

Case counts of abuse, misuse, and diversion were calculated for 1) Xtampza ER, 2) IR oxycodone, 3) other abuse-deterrent formulation (ADF) ER opioids, and 4) non-ADF ER opioids. The products included in each drug group, rationale for each group, prescriptions dispensed, and morphine equivalent grams dispensed are provided in Table 1.

**Table 1.** Drug groups included in analyses, rationale for inclusion, and dispensing data 2016-Q3 through 2019-Q2

Product Name	Rationale for Inclusion	Prescriptions,* Thousands	Morphine Equivalent Grams Dispensed,*Thousands
Xtampza ER	Target drug group	604	1,149
Other abuse-deterrent formulation extended-release opioid products	Other oxycodone, morphine, and hydrocodone products with labeling approved by the FDA that describes abuse-deterrent features	8,745	24,220
	Oxycontin	7,670	22,750
	Hysingla ER	593	687
	Embeda	399	659
	Morphabond ER	60	87
	Arymo ER	23	37
Non-abuse-deterrent formulation extended-release opioid products	Other marketed extended-release opioids that do not include labeling describing abuse-deterrent properties; limited to the same active pharmaceutical ingredients of abuse-deterrent opioids	16,274	35,664
	Nonbranded ER morphine	15,956	35,075
	Zohydro ER	236	328
	Kadian	62	168
	MS Contin	20	93
	Other products (Avinza)	<1	<1
Immediate-release oxycodone	Same active pharmaceutical ingredient as target drug but different formulation	132,091	158,702

ER = extended-release; FDA = Food and Drug Administration.

* Based on estimates provided by IQVIA (Danbury, CT, USA) US-Based Longitudinal Patient Data.

### Data Sources

The Poison Center Program obtains data from the general population seeking advice spontaneously after an exposure to a potentially toxic substance, including prescription opioids [8]. During the study period, the program included complete data from regional US poison centers that cover over 93% of the US population. For analyses, we created a composite *abuse/misuse* definition for cases by combining three exposure categories: *intentional abuse*, *intentional misuse*, and *intentional unknown* exposures as defined in the annual report of the National Poison Data System [14].

The Drug Diversion Program provides surveillance data on prescription drug diversion from municipal police departments, multijurisdictional drug task forces, county sheriffs’ departments, regulatory agencies (i.e., state medical and pharmacy boards), state police agencies, prosecutors’ offices, and departments of health [8]. At least 200 officers across 49 states and the District of Colombia (DC) submitted data each quarter on the number of documented drug diversion cases within their jurisdiction. A total of 272 agencies participated during the study period. A *diversion case* is defined by a participating agency as an instance of unlawful channeling of a product of interest from legal sources that results in a written report or complaint.

The Treatment Center Programs Combined refers to combined data from the Opioid Treatment Program and the Survey of Key Informant Patients Program. A new patient entering treatment in both programs is offered the opportunity to complete a standardized self-administered questionnaire that solicits information that identifies drugs the individual abused in the past month [8]. These programs share a similar questionnaire, and the data are often combined for analysis. The Opioid Treatment Program is composed of 69 participating methadone treatment programs (public and private) from 32 states [8]. The Survey of Key Informants’ Patients Program includes 126 substance abuse programs from 39 states. An *abuse case* was defined as a respondent who endorsed past-month use of one or more products within the drug groups of interest to get high by one of six routes of administration (injection, snorting, smoking, chewing, swallowing whole, dissolving in mouth).

The StreetRx Program uses the principle of crowdsourcing to collect and analyze black market drug price information via anonymous submissions to the StreetRx.com website. Site users spontaneously submit the street prices they paid, or heard were paid, for diverted prescription drugs. Users enter the name and dosage strength of the drug purchased, assisted by a structured list of controlled and noncontrolled substances. The site receives ∼4,200 price reports a month. Analyses were restricted to drug groups of interest.

The Web Monitoring Program combines qualitative and quantitative data collection methods. Data from posts on prescription drugs are collected using a Web crawling platform (Salesforce, San Francisco, CA, USA). The universe of public websites on the internet (>150,000,000 Web sites) was scraped to find online posts made in 2017 or 2018. For this analysis, manual searches of relevant discussion forums mentioning an opioid of interest were reviewed. To be included, a post had to have mentioned 1) a product name or 2) formulation release type. Liquids were not included. Posts were reviewed to determine route of administration. Posts were reviewed to determine whether respondents tampered with the medication. Posts were also reviewed for sentiment, which was defined as the dominating view or opinion of a drug within the post. Trained reviewers categorize posts as negative, positive, or neutral sentiment based on a standard protocol.

Information on drug utilization was obtained from the IQVIA (Danbury, CT, USA) US-Based Longitudinal Patient Data database. Drug utilization–adjusted estimates account for different degrees of exposure to prescription opioid products. This allows for comparisons of the expected number of cases or expected prevalence at equivalent levels of exposure. We selected two measures of exposure: prescriptions dispensed and morphine equivalent grams dispensed. The rate calculated with the prescriptions dispensed denominator provides an estimate of cases relative to the number of prescriptions filled at retail pharmacies. Morphine equivalent grams dispensed was chosen because drug groups compared within this study are not comparable in strength, potency, or tablets dispensed as part of a typical prescription. Misuse behaviors differ by these factors. Individuals with opioid use disorder were more likely to report consuming multiple pills with lower strength and lower-potency pills [15]. Using measures such as tablets dispensed may underestimate the abuse liability of IR opioids or Xtampza ER relative to higher-dose ER opioids because the number of tablets consumed per abuse event may vary due to differences in tablet strength. To provide an assessment of risk relative to the amount and potency of active pharmaceutical ingredient dispensed by drug group, rates were also adjusted for morphine equivalent grams dispensed [16]. Total grams dispensed for morphine and hydrocodone was used for morphine equivalent analyses since the conversion factor is 1. For oxycodone, grams dispensed were multiplied by the morphine equivalent adjustment factor of 1.5. Xtampza ER milligrams dispensed were converted to oxycodone HCl equivalents (e.g., 9 mg was converted 10 mg).

### Data Analysis

Poisson regression was used to compare cases for Xtampza ER and comparators in the Poison Center and Drug Diversion Programs adjusted for drug utilization. This analysis compares the expected number of cases between drug groups at the same utilization value. Logistic regression was used to compare odds of endorsement (cases relative to noncases) adjusted for drug utilization in the Treatment Center Programs Combined. This analysis compares the expected odds of an endorsement between drug groups at the same utilization value. Regression models were performed by program and by utilization denominator. In all models, utilization entered the model as an offset variable. For the Poison Center and Drug Diversion Programs, utilization offsets were calculated using the coverage of participating centers or agencies. As Treatment Center Programs Combined estimates were adjusted for quarterly changes in respondents, national utilization estimates were used. The model included a predictor variable for drug group with Xtampza ER as the reference category.

Price data from the StreetRx Program were assessed using univariate and multivariable linear regression. Because price data from the StreetRx Program were positively skewed, log price per milligram was used in the analyses. Both adjusted and unadjusted geometric mean prices are presented. Covariates included in the multivariable analysis are active pharmaceutical ingredient and dosage strength in milligrams; both are shown to be associated with street price [17, 18] and could confound the association between drug group and price per milligram.

In the Poison Center Program and Treatment Center Programs Combined, the percentage of cases where the product name and formulation were not known exceeded 10%. Missing data patterns were different across active pharmaceutical ingredient, with morphine having the largest percentage of missing information on formulation and product. Amioka and colleagues [19] observed that failing to account for missing information on product and formulation results in biased estimates when comparing cases between drug groups in the Poison Center Program. While all drug groups were underestimated, formulations within active pharmaceutical ingredients with the largest percentage of missing values were the most underestimated [19].

To address this limitation, multiple imputation [20], a standard method for analyzing data that are missing conditional on observed variables, was employed. Multiple imputation substitutes missing data with plausible values based on observed variables. For this analysis, separate imputation models were performed for each active pharmaceutical ingredient (hydrocodone, morphine, and oxycodone) due to large differences in the proportion missing. Within each active pharmaceutical ingredient, cases where the formulation was unknown were imputed into drug categories. Within oxycodone, missing cases were imputed into one of four drug groups: 1) Xtampza ER, 2) OxyContin, 3) ER opioid not identified as Xtampza ER or OxyContin, or 4) IR oxycodone. Within morphine, cases were imputed into one of five drug groups: 1) ADF-labeled single-entity ER morphine, 2) ADF-labeled combination morphine-naloxone ER tablets/capsules, 3) cases not identified as ADF ER morphine tablets/capsules, 4) IR morphine tablets/capsules, or 5) other morphine formulations (e.g., solutions). Within hydrocodone, cases could be imputed into one of five drug groups: 1) ADF ER hydrocodone, 2) non-ADF ER hydrocodone analgesic tablets, 3) ER hydrocodone cough/cold tablets, 4) IR hydrocodone, or 5) other hydrocodone formulations. A separate imputation model was run for each active pharmaceutical ingredient; 50 imputations were performed for each active pharmaceutical ingredient. The low case counts for some drug groups increased the likelihood of unstable models; therefore, no predictor or auxiliary variables were included in the imputation model. Therefore, the imputation of missing drug group values was informed by the observed proportions within each active pharmaceutical ingredient, with variations across data sets accounting for error in the assignment. Because outcome variables were nominal, fully conditional specification within the PROC MI SAS procedure was used [21]. After imputation into the above categories, cases were assigned to the appropriate drug group (Xtampza ER, other ADF ER opioids, non-ADF ER opioids, or IR oxycodone) for analyses. Models comparing rates or odds were performed across the 50 imputed data sets and combined using Rubin’s Rules [20]. Analyses were conducted using SAS (version 9.4; SAS Institute Inc., Cary, NC, USA). The Poison Center Program, Web Monitoring Program, and Opioid Treatment Program are approved by the Colorado Multiple Institutional Review Board (IRB). The Poison Center Program is approved by the IRB of each participating poison center. The Survey of Key Informants’ Patients Program is approved by the Washington University in St. Louis IRB. The protocol for the Drug Diversion Program was reviewed by the Nova Southeastern University IRB and determined to be non–human subject research and granted exempt status.

## Results

### Overall Misuse/Abuse/Diversion

**Figure 1.**
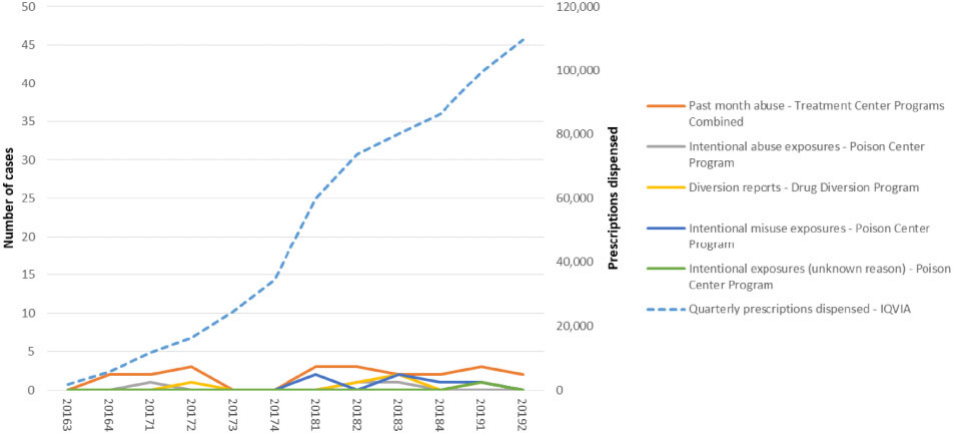
Xtampza extended-release abuse, misuse, and diversion by program and prescriptions dispensed by quarter.

The number of prescriptions dispensed quarterly for Xtampza ER increased from 1,876 in the second quarter of 2016 to 109,624 in the second quarter of 2019, a 58-fold increase (Figure 1). During this time, the Poison Center program received a total of 10 cases involving Xtampza ER (Table 2): three intentional abuse exposures, six intentional misuse cases, and one intentional unknown case. In the Treatment Center Programs Combined, there were 21 respondents who endorsed past-month abuse of Xtampza ER. In the Drug Diversion Program, there were five cases involving diversion of Xtampza ER reported. In contrast, IR oxycodone had more cases: Poison Center Program 5,292 cases, Treatment Center Program 4,113 endorsements, Drug Diversion Program 4,360 cases (Table 2).

**Table 2.** Cumulative abuse and misuse cases by program, drug group, and route of administration, 2016-Q3 through 2019-Q2

Drug Group	Poison Center Program, Total Intentional Abuse/Misuse/Unknown Exposures			Treatment Center Programs Combined, Past-Month Abuse			Drug Diversion Program, Total Events
	Cases, No.	Cases Involving Injection, No. (%)	Cases Involving Inhalation, No. (%)	Cases, No.	Cases Reporting Injection, No. (%)	Cases Reporting Snorting, No. (%)	Cases, No.
Xtampza ER	10	0 (0)	0 (0)	21	2 (9.5)	3 (14.3)	5
IR oxycodone	5,292	59 (1.1)	307 (5.8)	4,113	473 (11.5)	1,550 (37.7)	4,360
Other ADF ER opioids	817	31 (3.8)	76 (9.3)	2,158	343 (15.9)	672 (31.1)	313
Non-ADF ER opioids	486	9 (1.9)	10 (2.1)	628	185 (29.5)	113 (18.0)	418

ADF = abuse-deterrent formulation; ER = extended-release formulation; IR = immediate-release formulation.

**Figure 2.**
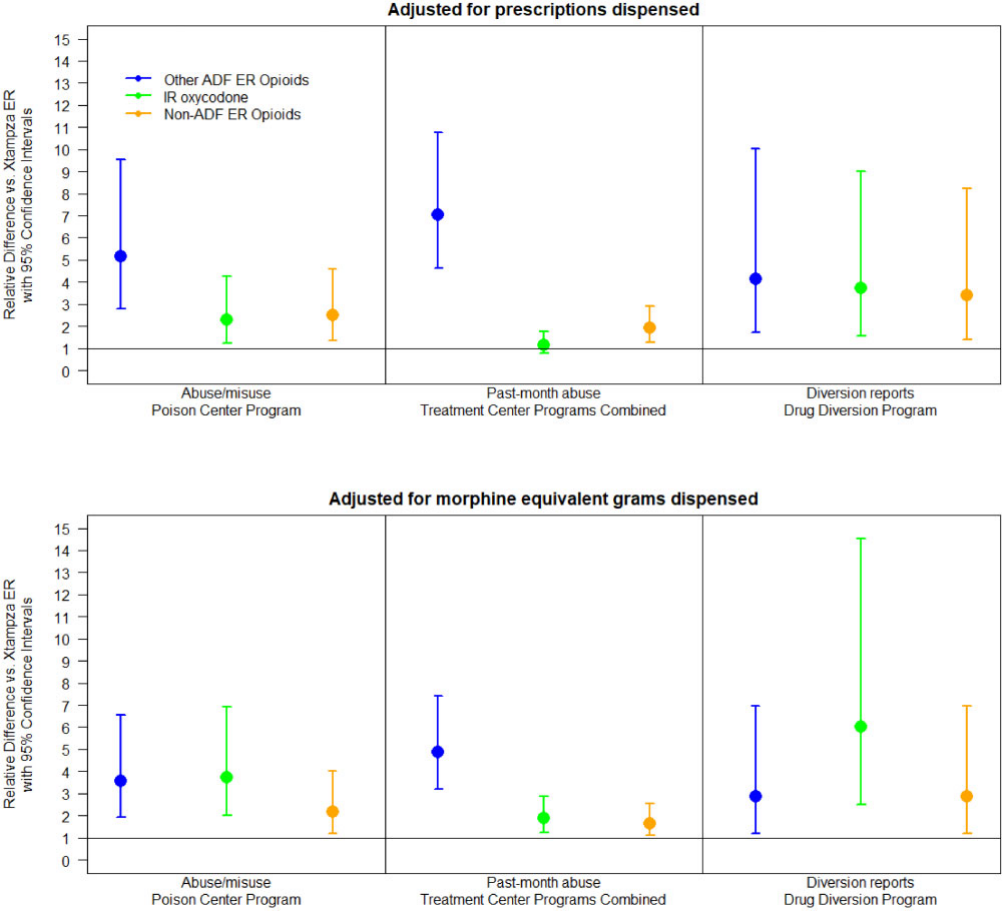
Relative difference with 95% confidence intervals between Xtampza extended-release and comparator drug groups by utilization measure and program.

The rate ratio of each drug group relative to Xtampza ER by program and utilization denominator is displayed in Figure 2. In the Poison Center Program, the rate ratio of abuse/misuse cases per prescription dispensed relative to Xtampza ER was greater for all three drug groups: IR oxycodone 2.3 (95% CI = 1.2–4.3, *P* = 0.008), other ADF ER opioids 5.2 (95% CI = 2.8–9.5, *P* < 0.001), and non-ADF ER opioids 2.5 (95% CI = 1.3–4.6, *P* = 0.004). Results were also statistically significant when adjusting for morphine equivalent grams dispensed: IR oxycodone 3.7 (95% CI = 2.0–6.9, *P* < 0.001), other ADF ER opioids 3.6 (95% CI = 1.9–6.6, *P* < 0.001), and non-ADF opioids 2.2 (95% CI = 1.2–4.0, *P* = 0.014).

In the Treatment Center Programs Combined, 21 (0.09%) of 22,793 valid surveys endorsed using Xtampza ER to get high (Table 2). Compared with Xtampza ER, the odds ratio of a respondent endorsing past-month abuse was higher: other ADF ER opioids 7.4 (95% CI = 4.8–11.4, *P* < 0.001) and non-ADF ER opioids 2.0 (95% CI = 1.3–3.1, *P* = 0.002) (Figure 2). The difference with IR oxycodone did not achieve statistical significance (OR = 1.2, 95% CI = 0.8–1.9, *P* = 0.349). After adjusting for morphine equivalent grams dispensed, all three groups were greater than Xtampza ER: IR oxycodone 1.9 (95% CI = 1.3–3.0, *P* = 0.003), other ADF ER opioids 5.1 (95% CI = 3.3–7.8, *P* < 0.001), and non-ADF ER opioids 1.7 (95% CI = 1.1–2.7, *P* = 0.012).

There were five reports in the Diversion Program involving Xtampza ER (Table 2). In the Drug Diversion Program, the rate ratios using prescriptions dispensed were consistently higher compared with Xtampza ER: IR oxycodone 3.7 (95% CI = 1.6–9.0, *P* = 0.003), other ADF ER opioids 4.1 (95% CI = 1.7–10.0, *P* = 0.002), non-ADF ER opioids 3.4 (95% CI = 1.4–8.2, *P* = 0.007) (Figure 2). Adjusting for morphine equivalent grams dispensed, the rate ratio for IR oxycodone was greater than Xtampza ER: 6.0 (95% CI = 2.5–14.4, *P* < 0.001); other ADF ER opioids 2.9 (95% CI = 1.2–6.9, *P* = 0.019), and non-ADF ER opioids 2.9 (1.2–6.9, *P* = 0.019) (Figure 2).

### Route of Administration

In the Poison Center Program, no abuse/misuse cases involved use of Xtampza ER by injection or inhalation. By contrast, inhalation and injection were reported for all comparator groups (Table 2). In the Treatment Center Program, 21 respondents endorsed Xtampza ER abuse; two (9.5%) endorsed use by injection, and three (14.3%) endorsed use by snorting. Of the two respondents who reported injection use, both additionally reported injection use of other ER oxycodone (one of these subjects reported injection of every ER oxycodone listed on the response sheet, including products not currently marketed). One respondent also reported injection use of oxymorphone. Of the three respondents who reported snorting Xtampza ER, all reported snorting other oxycodone products as well. One respondent also reported snorting oxymorphone and morphine, and another reported snorting heroin.

### Street Price

Overall, the unadjusted geometric mean price of oxycodone IR was highest (Table 3). There are known influences on street price by tablet strength (price per milligram decreases as tablet content increases) [18] and active pharmaceutical ingredient (API) potency (price increases as potency increases) [17]. After adjusting for these factors, oxycodone IR had the highest price and Xtampza ER the lowest price; however, the geometric mean street price among groups was not statistically different (Table 3).

**Table 3.** Geometric mean price per milligram by drug group and API, unadjusted and adjusted ratio of geometric mean prices per milligram

Value	Value	No.	Geometric Mean Price per mg	Unadjusted Ratio of Geometric Mean Price per mg	Adjusted Ratio of Geometric Mean Price per mg^a^
			(95% CI)	(95% CI)	(95% CI)
Drug Group	Xtampza ER	157	$0.59 ($0.51–$0.69)	Ref	Ref
	IR oxycodone	9,027	$0.99 ($0.97–$1.01)	1.67 (1.43–1.94), *P* < 0.001	1.08 (0.93–1.25), *P* = 0.335
	Other ADF ER opioids	2,012	$0.50 ($0.48–$0.52)	0.84 (0.72–0.98), *P* = 0.030	1.11 (0.95–1.29), *P* = 0.176
	Non-ADF ER opioids	745	$0.33 ($0.31–$0.35)	0.59 (0.51–0.69), *P* < 0.001	1.18 (0.97–1.45), *P* = 0.103
API	Oxycodone	10,729	$0.90 ($0.89–$0.92)	Ref	Ref
	Hydrocodone	503	$0.37 ($0.34–$0.40)	0.41 (0.37–0.44), *P* < 0.001	0.69 (0.63–0.77), *P* < 0.001
	Morphine	709	$0.31 ($0.29–$0.33)	0.34 (0.32–0.37), *P* < 0.001	0.57 (0.50–0.66), *P* < 0.001
mg strength	Natural log of mg strength		—	0.59 (0.58–0.61), *P* < 0.001	0.63 (0.61–0.65), *P* < 0.001

ADF = abuse-deterrent formulation; API = active pharmaceutical ingredient; ER = extended-release formulation; IR = immediate-release formulation.

* Drug group ratios are adjusted for variables associated with price per milligram, specifically active pharmaceutical ingredient and pill dosage strength in milligrams.

### Online Reports of Tampering and Sentiment

There was a total of 15 posts where authors described materials and methods they used in efforts to overcome the abuse-deterrent properties of Xtampza ER. All reports for Xtampza ER described attempts to overcome the delayed-release properties. Ten of the methods produced a form intended to be administered orally, and five described methods to create a substance to be taken via insufflation. A method was determined to be “effective” based on the author’s comments.

Of the 10 posts describing oral administration, six discussed a variety of methods to dissolve Xtampza ER capsules in a liquid with certain physical characteristics for an extended period of time (all exceeded four hours) and swallowing the contents. In five of the six posts, the method was judged by the writer to be effective. In one post, the author reported that the method was not effective. Three posts contained an expansion of this method of by further processing Xtampza ER. Another approach described heating and melting the microspheres and swallowing the substance, which the post author described as ineffective. In each case, the ultimate outcome was a solution for oral administration.

Of the five methods addressing intranasal administration, four posts described physical manipulation including pretreatment of the Xtampza ER microspheres and combining with other substances to create a powder. Two authors reported that these approaches were effective; in two other posts, the effectiveness was unclear. The authors reported substantial discomfort in taking the new form created intranasally. Another approach involved attempting to simply crush the microspheres and snorting the results, which the author judged to be ineffective.

We also examined case notes from the 10 intentional exposures to Xtampza ER from the Poison Center Program. These notes are recorded in real time during case management by trained nurse or pharmacist specialists in poison information. All cases involved ingesting multiple tablets; none described manipulation methods found online.

Results for sentiment included a total of 362 posts for Xtampza ER, an estimated 261 posts for oxycodone IR, an estimated 244,941 posts for other ADF ER opioid products, and an estimated 530 posts for non-ADF ER opioid products. Xtampza ER had the lowest proportion of posts that encouraged unsafe or inappropriate use of the drug (Table 4).

**Table 4.** Sentiment of Web monitoring posts for opioid analgesics

Drug Group	Observations, No.	Positive (95% CI)	Neutral (95% CI)	Negative (95% CI)
Xtampza ER	362	11.6	69.6	18.8
Oxycodone IR	33	3.2 (0.0–9.2)	69.6 (53.9–85.3)	27.3 (12.1–42.4)
Other ADF ER	1,304	2.7 (1.8–3.6)	45.0 (42.2–47.7)	52.3 (49.6–55.0)
Non-ADF ER	105	19.7 (11.7–27.6)	50.1 (40.1–60.2)	29.6 (20.4–38.8)

All posts from Xtampza ER were coded; therefore, CIs are not provided. Due to the large volume, posts for comparators were sampled randomly before coding; estimates and 95% CIs of total numbers of posts were calculated. Positive sentiment promotes the safe use or therapeutic benefits of the drug. Negative sentiment encourages unsafe or inappropriate use of the drug or report of ineffectiveness or side effects of the drug. Neutral sentiment makes no reference to either a positive or negative sentiment, or the sentiment cannot be determined.

ADF = abuse-deterrent formulation; ER = extended-release formulation; IR = immediate-release formulation.

## Discussion

Results from users contacting a poison center who were entering treatment for substance abuse and drug diversion programs indicate that oxycodone IR had the highest number of abuse, misuse, and diversion cases. The number of Xtampza ER prescriptions dispensed per quarter increased more than 50-fold during the same period, but the number of cases in each program was low and remained infrequent throughout the study period. This finding is notable given previously reported trends with opioid prescribing and misuse. Alturi and colleagues note that as grams of oxycodone dispensed increased 117% between 2004 and 2011, emergency department visits involving misuse of oxycodone increased 263% [22]. The Centers for Disease Control and Prevention reported that between 1999 and 2008, sales of opioid pain relievers increased fourfold while treatment admissions involving opioid pain reliever abuse of dependence increased sixfold [23].

After adjusting for two different drug availability measures (prescriptions dispensed and morphine equivalent milligrams dispensed), abuse, misuse, and diversion of both other ADF ER opioids and non-ADF ER opioids was significantly greater than Xtampza ER. IR oxycodone showed a slightly different pattern. In the Treatment Center Programs Combined, the prevalence of past-month abuse of Xtampza ER among individuals entering treatment for opioid use disorders was not different from IR oxycodone when adjusted for prescriptions dispensed. Interestingly, the prevalence of IR oxycodone use was again significantly greater than Xtampza after adjustment using morphine equivalent grams dispensed. This observation may be the result of large prescription volumes with a small number of tablets, characteristic of lower-dosage oxycodone. For example, oxycodone 5 mg is often prescribed for short-duration illness in patients with low abuse potential.

The results from Web monitoring are consistent with the other programs. The proportion of posts encouraging unsafe or inappropriate use of the drug or reporting ineffectiveness or side effects was lowest for Xtampza ER. Detailed analysis of posts showed that most manipulated Xtampza ER to improve oral abuse, which is a less dangerous route of exposure than intranasal or intravenous abuse. All comparator groups had higher rates of intranasal and intravenous abuse.

Xtampza ER was approved with labeling for abuse deterrence by the oral, intravenous, and intranasal routes. All three routes show low rates of abuse and did not increase during the study period. The low frequency of Xtampza ER use by nonoral routes requires remark. In the Poison Center Program, there were no cases involving inhalation or intravenous abuse, which captures information from the individual or their care provider at the time of the incident. Consistent with poison center data, Web discussion boards and forums suggest that some methods of tampering with Xtampza ER were explored by users; however, the frequency was very low, and none described use via injection. The Treatment Center Program represents more experienced and higher-intensity users. Some inhalational and injection use of Xtampza ER was endorsed. However, the proportion of past-month abuse cases involving the injection and inhalation routes of administration was lower for Xtampza ER than oxycodone IR, other ADF ER opioids, and non-ADF ER opioids.

One challenge with survey data such as data from the Treatment Center Program is errant responses. With infrequent endorsements that are not increasing as prescription volume increases, it is difficult to determine whether these cases are an indication of a rare behavior or simply errant product endorsements. A limitation is that these rare events may be overestimated. Continued monitoring and follow-up of individuals reporting unintended routes of abuse of Xtampza ER are warranted to determine if these endorsements are due to random error or an indication of an emerging abuse behavior.

Differences in the unadjusted street price were observed, but these differences failed to achieve statistical significance after adjustment for tablet size and API. Further investigation into this observation is warranted, particularly in relation to comparing products at different strengths using street price data.

There are limitations to this study. The Poison Center and Treatment Center Program cases involve self-report. Differential misidentification among drug groups may affect observed differences. Case counts of drug groups that were comprised primarily of branded products (other ADF ER opioids) may be overestimated when based on self-report, and drug groups that were comprised primarily of generic products (non-ADF ER opioids and IR oxycodone) may be underestimated [24].

The study methodology also has notable strengths. These data sources include specific drug identities, which are often incomplete or not available in other data sources. In addition, all programs included in this analysis began surveillance of Xtampza ER with product launch in June 2016. This analysis accounted for unknown formulations within active pharmaceutical ingredient, reducing potential bias in comparisons between drug groups. Poison center cases are sensitive to changes in prescription opioid abuse and show strong associations with overdose deaths [25]. Treatment center data surveys obtain information from a hard-to-reach, vulnerable population that engages in high-risk behaviors. This population has been shown to be sensitive to changes in unintended routes of abuse following introduction of tamper-resistant formulations [26]. Diversion and StreetRx provide product-specific information on illegal distribution channels, and the Web Monitoring Program allows for continuous evaluation of methods used to defeat tamper-resistant properties of opioid analgesics.

## Conclusions

Abuse, misuse, and diversion of Xtampza ER have remained low compared with commonly abused Schedule II opioid analgesics for three years after introduction into the US market. Methods to defeat the tamper-resistant properties of Xtampza ER have been reported, but there is no indication of widespread or expanding abuse or misuse in the data streams evaluated. As use of Xtampza ER increases, more drug will be available for misuse; continued monitoring is warranted.

## Notes

Funding sources: The RADARS System is supported by subscriptions from pharmaceutical manufacturers and government and nongovernment agencies for surveillance, research, and reporting services. The RADARS System is the property of Denver Health and Hospital Authority, a political subdivision of the State of Colorado. Denver Health retains exclusive ownership of all data, databases, and systems. Subscribers do not participate in data collection, nor do they have access to the raw data. This research was sponsored by Collegium Pharmaceutical. Xtampza ER is a registered trademark of Collegium Pharmaceutical.
